# Cerebral Venous Infarction Due to Deep Medullary Venous Thrombosis in a Neonate with Severe Milk-induced Non-IgE-mediated Gastrointestinal Food Allergy: A Case Report

**DOI:** 10.31662/jmaj.2025-0214

**Published:** 2025-09-05

**Authors:** Chisato Jimbo, Kiwako Yamamoto-Hanada, Kouhei Hagino, Daichi Suzuki, Tomoki Yaguchi, Daisuke Harama, Marei Omori, Kotaro Umezawa, Fumi Ishikawa, Seiko Hirai, Kenji Toyokuni, Shoji Mizuno, Akihiro Iguchi, Reiko Okamoto, Shotaro Matsumoto, Ichiro Nomura, Tatsuki Fukuie

**Affiliations:** 1 Allergy Center, National Center for Child Health and Development, Tokyo, Japan; 2 Division of Hematology, National Center for Child Health and Development, Tokyo, Japan; 3 Department of Radiology, National Center for Child Health and Development, Tokyo, Japan; 4 Division of Critical Care Medicine, National Center for Child Health and Development, Tokyo, Japan

**Keywords:** non-IgE-mediated gastrointestinal food allergies, food protein-induced enterocolitis syndrome, cerebral infarction

## Abstract

Cow’s milk-induced non-IgE-mediated gastrointestinal food allergies (non-IgE-GIFAs) are common and generally considered to have a favorable prognosis; however, severe cases can occur. We report a case of a neonate who presented with hypovolemic shock and subsequently developed cerebral infarction due to deep medullary venous thrombosis. A female infant, born at 35 weeks of gestation, was initially fed formula but transitioned to exclusive breastfeeding within a few days. After discharge on day 17, mixed feeding was resumed, leading to frequent pale-colored diarrhea. On day 19, she developed hypovolemic shock and was admitted to the intensive care unit. Her symptoms improved with nil per os management; however, on day 20, her hemoglobin levels declined despite no signs of bleeding on ultrasonography. She was treated with red blood cell transfusion and intravenous vitamin K. An amino acid-based formula was introduced on day 22 without adverse events, which suggested a possibility of non-IgE-GIFAs. However, the patient developed intermittent transient fevers thereafter; a cranial ultrasound performed on day 41 revealed a low-echoic area in the right frontal lobe. Subsequent magnetic resonance imaging revealed a cystic lesion in the right frontal lobe accompanied by surrounding parenchymal degeneration, leading to a diagnosis of venous infarction due to deep medullary vein thrombosis. Blood tests ruled out vitamin K deficiency and protein C or S defects. Despite this neurologic complication, she exhibited no neurologic symptoms throughout the clinical course. At 10 months of age, her neurodevelopment remains normal without any delays. This case highlights the potential for severe complications, including cerebral venous infarction, in neonatal non-IgE-GIFAs. Close monitoring for systemic complications is crucial in severe cases.

## Introduction

Non-IgE-mediated gastrointestinal food allergies (non-IgE-GIFAs) have been increasingly recognized in recent years. A nationwide survey in Japan reported an estimated incidence of 0.20%, with almost a half of cases occurring during the neonatal period ^(1)^. Although the prognosis of cow’s milk-induced non-IgE-GIFAs is generally considered favorable, some early-onset cases can present with severe symptoms ^(1), (2), (3), (4)^. Here, we report a rare case of suspected neonatal non-IgE-GIFAs presenting with hypovolemic shock, followed by cerebral venous infarction due to deep medullary venous thrombosis (DMVT).

## Case Report

**Figure 1.**
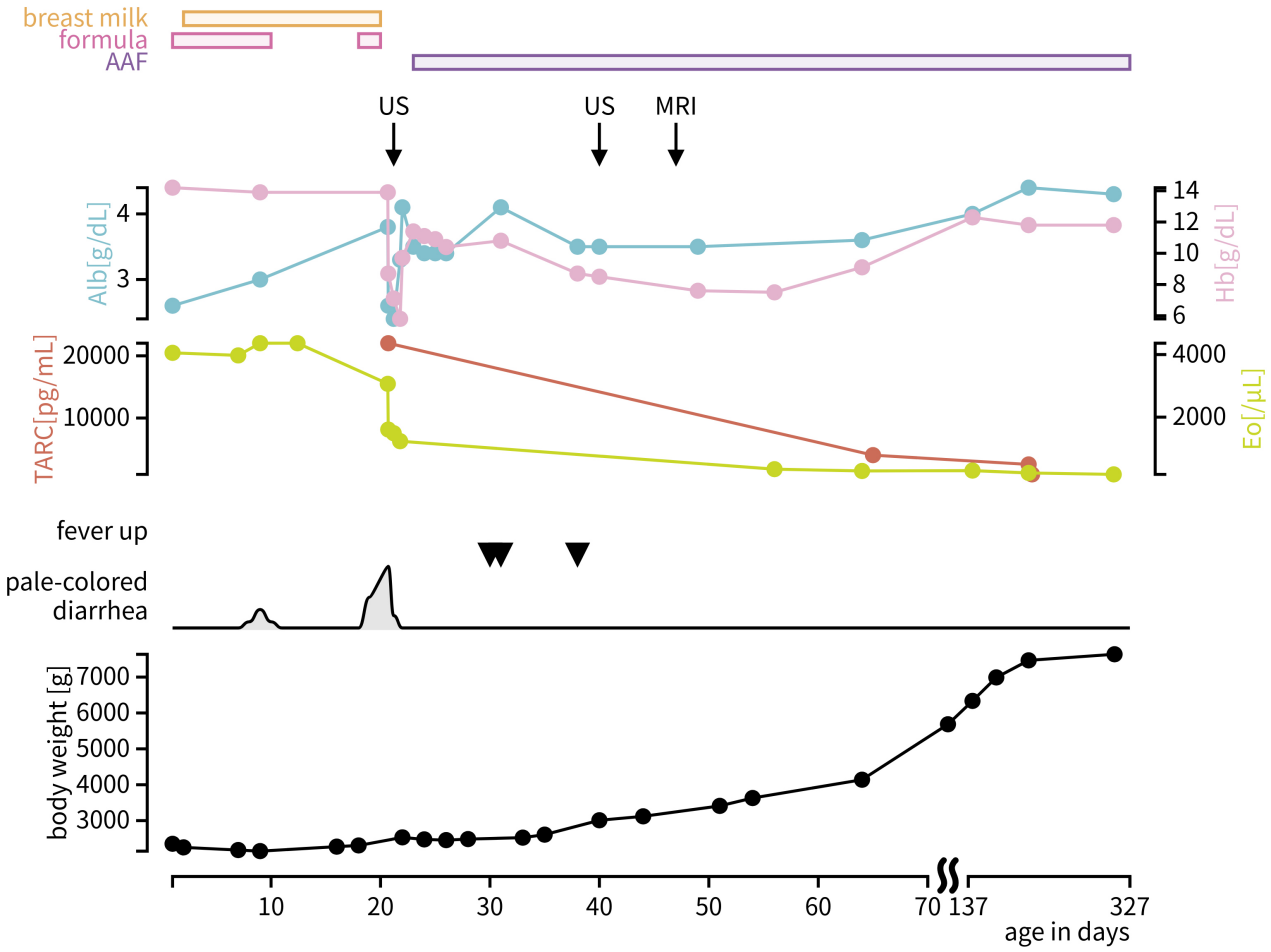
Clinical course. AAF: amino acid--based formula; Alb: albumin; Eo: eosinophil counts; Hb: hemoglobin; MRI: magnetic resonance imaging; TARC: thymus and activation-regulated chemokine; US: ultrasonography.

The patient was a female neonate, 19 days old at presentation. She was born at 35 weeks and 0 days of gestation by cesarean delivery, with a birth weight of 2,354 g. She was initially fed mainly with formula during the first several days and experienced weight loss until day 11. Feeding was then transitioned to near-exclusive breastfeeding within 7-10 days, after which she began gaining weight at a rate of 17 grams per day. On day 8, transient pale stools were observed; however, no findings suggestive of biliary atresia were noted, and the condition resolved spontaneously within a few days. After being discharged on day 17, formula feeding was reintroduced, after which she developed frequent pale-colored diarrhea and hypovolemic shock, necessitating admission to our intensive care unit (ICU) (Figure 1, Table 1).

**Table 1.** Results of Blood and Stool Examination on Day 1.

CBC					Biochemical				VBG		
WBC		14850	/μL		T-Bil	8.83	mg/dL		pH	7.258	
	Neu	30.2	%		D-Bil	0.78	mg/dL		pCO2	21.5	mmHg
	Lym	48.2	%		AST	32	U/L		tHb	8.8	g/dL
	Eos	10.8	%		ALT	85	U/L		MetHb	3.2	%
RBC		247	10*4/μL		LDH	487	U/L		Lac	7.4	mmol/L
Ht		25.2	%		γGTP	71	U/L		B.E.	−16.3	mmol/L
Hb		8.7	g/dL		ChE	167	U/L				
Plt		37.2	10*4/μL		TP	3.9	g/dL				
					Alb	2.6	g/dL		**Special assays**		
**Coagulation**					BUN	26	mg/dL		TARC	22030	pg/mL
PT-INR		1.64			Cre	0.62	mg/dL				
APTT		43.2	sec		UA	11	mg/dL				
ATⅢ		52.3	%		Na	146	mEq/L		**Stool viral antigen**		
D-dimer		1.1	μg/mL		Cl	116	mEq/L		Rotavirus	-	
fibrinogen		205	mg/dL		K	4.6	mEq/L		Adenovirus	-	
					Mg	2.4	mg/dL				
					CK	175	U/L				
					Amy	≤3	U/L				
					CRP	0.54	mg/dL				

Alb: albumin; ALT: alanine transaminase; Amy: amylase; APTT: activated partial thromboplastin time; AST: aspartate transaminase; ATIII: antithrombin III; B.E.: base excess; BUN: blood urea nitrogen; CBC: complete blood count; ChE: cholinesterase; CK: creatine kinase; Cl: chlorine; Cre: creatinine; CRP: C reactive protein; D-Bil: direct bilirubin; γGTP: gamma-glutamyl transferase; Hb: hemoglobin; Ht: hematocrit; K: potassium; Lac: lactate; LDH: lactate dehydrogenase; MatHb: methemoglobin; Mg: magnesium; Na: sodium; Plt: platelet; PT-INR: prothrombin time/international normalized ratio; T-Bil: total bilirubin; TARC: thymus and activation-regulated chemokine; tHb: total hemoglobin; TP: total protein; UA: uric acid; VBG: Venous Blood Gas; WBC: white blood cell.

**Figure 2.**
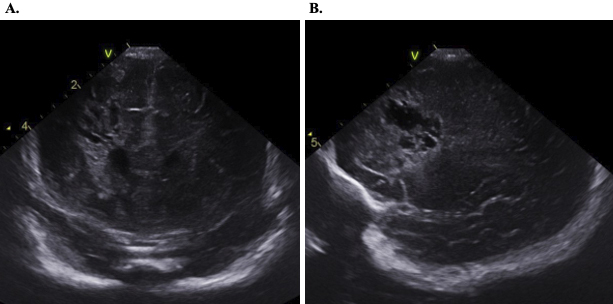
Intracranial ultrasonography. Ultrasonography on day 41 showed a cystic change with a surrounding hyperechoic around the right frontal horn of the lateral ventricle. Coronal (A) and sagittal views (B) are shown.

**Figure 3.**
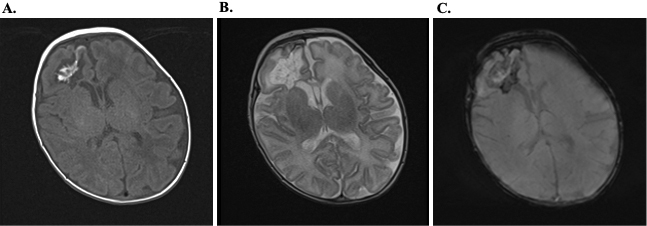
Head MRI. Head magnetic resonance imaging on day 46 showed DMVT and associated peripheral post-infarction changes, such as cystic encephalomalacia, confirming cerebral venous infarction. Axial T1-weighted images demonstrate linear hyperintense lesions in right frontal lobe (A). The corresponding T2-weighted image shows linear hypointense alterations at the same location (B). Susceptibility-weighted imaging did not show any hypointense signals in the affected area, suggesting a thrombotic rather than hemorrhagic process (C). These linear signal changes are suggestive of DMVT. Cystic encephalomalacia is observed in the surrounding parenchyma, indicating secondary tissue degeneration. No thrombosis was observed in the cerebral venous sinuses. DMVT: deep medullary venous thrombosis; MRI: magnetic resonance imaging.

Her condition stabilized with systemic management, including vasopressors, intravenous fluids, and bowel rest (nil per os). The next day, her hemoglobin levels dropped despite no evidence of bleeding on intracranial nor abdominal ultrasonography. Red blood cell transfusion and intravenous vitamin K were administered, with no further deterioration. An amino acid-based formula was introduced on day 22 without adverse reactions, leading to a suspicion of non-IgE-GIFAs. Her general condition remained stable after discharge from the ICU. However, transient fever occurred on days 30, 31, and 38, in the absence of other symptoms. A cranial ultrasound conducted as part of screening identified a low-echoic cystic lesion with surrounding high-echoic region in the right frontal lobe (Figure 2). Head magnetic resonance imaging on day 46 demonstrated findings consistent with DMVT and associated peripheral post-infarction changes, confirming cerebral venous infarction (Figure 3). No vascular malformations were identified in magnetic resonance angiography. Blood investigations ruled out vitamin K deficiency, protein C or S defects, and autoimmune conditions such as antiphospholipid syndrome or systemic lupus erythematosus (Table 2). Throughout the clinical course, she exhibited no neurologic symptoms and was discharged on day 56. At 11 months of age, her neurodevelopment remains within normal limits without any delays.

**Table 2.** Results of Other Blood Examination.

Coagulation				Autoantibody panel				FOBT	
Protein C Activity	39	％		Antiphospholipid Antibody Panel				Hemoglobin	≤20
Protein C Antigen	33	％		Anti-CL-IgG	3	U/mL		Transferrin	≤5
Protein S Activity	67	%		Anti-CL-IgM	<1.0	U/mL			
Protein S Antigen	52	%		Anti-β2GP1-IgG	<6.4	U/mL			
				Anti-β2GP1-IgM	<1.1	U/mL			
**Iron Studies**				SCT Screening Value	36.1				
Ret%	7.3	%		SCT Confirmatory Value	69.6				
Iron	69	μg/dL		SCT Ratio	0.49				
TIBC	240	μg/dL							
UIBC	171	μg/dL		**Allergic assay**					
				Total IgE	2.5	IU/mL			
Vitamins				Milk-specific IgE	<0.1	UA/mL			
PIVKA-2	24	mAU/mL		Casein-specific IgE	<0.1	UA/mL			

CL: cardiolipin; FOBT: Fecal Occult Blood Test; IgG: immunoglobulin G; IgM: immunoglobulin M; PIVKA-2: protein induced by vitamin K absence-II; SCT: silica clotting time; TIBC: total iron binding capacity; UIBC: unsaturated iron binding capacity; β2GP1: beta-2-glycoprotein 1.

## Discussion

This is a rare case of neonatal non-IgE-GIFAs complicated by cerebral venous infarction due to DMVT after hypovolemic shock.

To the best of our knowledge, this is the first report describing early-onset non-IgE-GIFAs, followed by DMVT. Repetto et al. ^(5)^ reported a single case of cerebral sinovenous thrombosis (CSVT) associated with iron deficiency anemia secondary to chronic non-IgE-GIFAs.

Cerebral infarction is more common in neonates than in older children, with venous infarctions accounting for approximately 20% of cases; about half of these are CSVT ^(6), (7)^. DMVT is particularly rare. Venous infarctions are often caused by dehydration, and, in our case, severe diarrhea and subsequent hypovolemia can be a trigger. Chronic iron deficiency anemia may promote venous thrombosis via thrombocytosis; however, thrombocytosis was not observed in this patient ^(6)^. Although protein C and S levels were transiently low, they had normalized by 10 months of age, suggesting temporary reductions likely related to disseminated intravascular coagulation. Therefore, this case involved a neonate with no congenital risk for thrombosis, developing a venous infarction caused by non-IgE-GIFAs.

Although cerebral venous infarction typically presents with seizures, it can remain asymptomatic in neonates, as in this case ^(8)^. Moreover, although neonatal DMVT is often associated with long-term neurological sequelae, such as developmental delay or epilepsy, our patient has shown no such complications to date. As a limitation, neurodevelopmental assessment was performed not with a formal, internationally recognized test but using the Enjoji Analytical Developmental Test.

### Conclusion

We report a rare case of suspected neonatal non-IgE-GIFAs complicated by venous infarction due to DMVT. This case highlights the importance of close monitoring for systemic complications, such as cerebral infarction, in severe presentations of non-IgE-GIFAs.

## Article Information

### Acknowledgments

The author thanks all the present and previous members of the Pediatric Intensive Care Unit and Division of Neurology, National Center for Child Health and Development for their contributions to the work mentioned in this manuscript.

### Author Contributions

Chisato Jimbo and Kiwako Yamamoto-Hanada established the concept of this case study. All authors followed the case in the hospital. Kouhei Hagino, Daichi Suzuki, Tomoki Yaguchi, Marei Omori, Daisuke Harama, Kotaro Umezawa, Fumi Ishikawa, Seiko Hirai, and Kenji Toyokuni oversaw the patient’s care. Tatsuki Fukuie and Ichiro Nomura supervised this case. Shoji Mizuno and Akihiro Iguchi was responsible for the assessment of blood coagulation disorders. Reiko Okamoto conducted the evaluation of radiological imaging. Shotaro M. supervised the treatment in the pediatric intensive care unit. Chisato Jimbo wrote the first draft of the manuscript. All authors critically reviewed the manuscript and approved the final version.

### Conflicts of Interest

None

### IRB Approval Code and Name of the Institution

Not applicable. Informed consent was obtained verbally after thorough explanation, and this was documented in the clinical record.
